# Angiogenesis inhibitors in clinical development; where are we now and where are we going?

**DOI:** 10.1038/sj.bjc.6601401

**Published:** 2004-01-06

**Authors:** F A L M Eskens

**Affiliations:** 1 Department of Medical Oncology, Erasmus University Medical Center, PO Box 2040, Rotterdam 3000 CA, The Netherlands

**Keywords:** angiogenesis inhibitors, clinical trials

## Abstract

Angiogenesis is crucial for tumour growth and the formation of metastases. Various classes of angiogenesis inhibitors that are each able to inhibit one of the various steps of this complex process can be distinguished. Results from clinical studies with these agents are summarised. In general, it has been shown that most angiogenesis inhibitors can be safely administered, but that tumour regressions are rare. Combining angiogenesis inhibitors with cytotoxic chemotherapy can enhance anticancer activity. Recently, some promising data with regard to clinical efficacy have been presented. While performing clinical studies with angiogenesis inhibitors, defining biological activity is crucial, but thus far no validated techniques are available. It is conceivable that in the near future various classes of angiogenesis inhibitors will be combined in an attempt to further improve antiangiogenic and anticancer activity.

In order to grow and metastasise, tumours exceeding the size of 1 mm^3^ are dependent on blood supply from newly formed adjacent blood vessels.

Tumour-related angiogenesis is a multistep process that is initiated through the activity of various proangiogenic factors or stimuli that are secreted by tumour cells and host components such as macrophages, lymphocytes and kidney cells (Klagsbrun and D' Amore, 1996). Of these proangiogenic factors, vascular endothelial growth factor (VEGF) is predominant. Vascular endothelial growth factor binds with high affinity to the transmembrane endothelial VEGF receptor type 1 and 2 (VEGFR-1 or flt-1 and VEGFR-2 or FLK-1/KDR, respectively). Ligand–receptor interaction initiates a cascade of intracellular signals through increased receptor tyrosine kinase activity, resulting in endothelial cell proliferation and the formation of new blood vessels. Increased expression of VEGF is associated with poor clinical outcome irrespective of tumour stage or tumour grade.

Besides VEGF, basic fibroblast growth factor (bFGF), platelet-derived growth factor (PDGF), interleukin-8 and insulin-like growth factor (IGF) are factors involved in angiogenesis, and these factors have their own endothelial cell receptors.

As angiogenesis is of crucial importance for tumour growth, inhibiting this process has become a major challenge in the development of new anticancer agents.

Tumour-related angiogenesis is dependent on specific growth factors, the presence and activation of endothelial cell receptors, endothelial cell proliferative activity as such, and the interaction between proliferating endothelial cells and extracellular matrix components. All these specific steps can be inhibited by targeted agents.

Neutralising VEGF prior to its attachment to endothelial receptors can be accomplished by monoclonal antibodies. Inhibiting the tyrosine kinase activity of VEGFR-2, PDGF and/or bFGF receptors following ligand binding can be achieved through specific inhibitors. Inhibiting endothelial cell proliferation can be achieved by mimicking the activity of naturally occurring antiangiogenic proteins such as thrombospondin-1 (TSP-1), endostatin and angiostatin. Inhibiting the interaction between proliferating endothelial cells and various extracellular matrix components can be accomplished by inhibiting the activity of the transmembrane endothelial cell integrins *α*v*β*3 and *α*v*β*5. In contrast to quiescent blood vessels where expression of integins is minimal, actively proliferating endothelial cells express integrins to a high extent. Integrin activity can be inhibited by antibodies or small molecule inhibitors.

A different approach that may lead to diminished tumour vascularisation is the destruction of established vasculature. The so-called ligand-directed vascular targeting agents (VTAs) are able to induce a rapid and specific shutdown of tumour blood supply either by using antibodies, peptides or growth factors that deliver toxins and procoagulants or apoptotic effectors specifically to the tumour endothelium. The so-called small-molecule VTAs try to recognise the pathophysiological differences between the normal and tumour endothelium, and are thus able to induce vascular shutdown, specifically in tumours.

In this review, we will give an overview of the results of clinical studies that have been performed with various classes of antiangiogenic and antivascular agents (Table 1

**Table 1:** Angiogenesis inhibitors in clinical trials

**Drug**	**Company**	**Target**	**Clinical development**
*Monoclonal antibodies targeting VEGF-A*			
Bevacizumab (Avastin®)	Genentech	VEGF-A	Phase I, II, III
VEGF-Trap	Regeneron	VEGF-A	Phase I

*Antibodies targeting VEGFR-2*			
IMC-1C11	Imclone	VEGFR-2	Phase I

*Receptor tyrosine kinase inhibitors*			
SU5416	Sugen/Pharmacia	VEGFR-2	Phase I, II, III
SU6668	Sugen/Pharmacia	VEGFR-2, bFGFR, PDGFR	Phase I, II
SU11248	Sugen/Pharmacia	VEGFR-2, PDGFR,	
c-Kit, Flt-3	Phase I		
PTK787/ZK22854	Schering/Novartis	VEGFR-1, VEGFR-2	Phase I
ZD6474	Astra Zeneca	VEGFR-2, EGFR	Phase I, II
CP-547,632	Pfizer	VEGFR-2, EGFR, PDGFR	Phase I

*Inhibitors of endothelial cell proliferation*			
ABT-510	Abbott	Endothelial CD-36	Phase I, II
Angiostatin	Entremed	Various	Phase I
Endostatin	Entremed	Various	Phase I
TNP-470	TAP	Methionine aminopeptidase, cyclin dependent kinase 2	Phase I
Thalidomide	Grunenthal	Reduction of TNF-*α* production	Phase I, II, III

*Inhibitors of integrin activity*			
Vitaxin	MedImmune	Integrin *α*V*β*3	Phase I, II
Medi-522	MedImmune	Integrin *α*V*β*3	Phase I
Cilengitide	Merck KgaA	Integrin *α*V*β*3	Phase I

*Vascular targeting agents*			
Combretastatin A4	Oxigene	Endothelial tubulin	Phase I
AVE8062A	Aventis	Endothelial tubulin	Phase I
ZD6126	Astra Zeneca	Endothelial tubulin	Phase I
DMXAA		Induction of TNF-*α*	Phase I

). While discussing the results of these studies, we will focus in more detail on the mechanisms of action, and we will discuss some of the difficulties that have been faced while performing these studies. Finally, we will give some thoughts on where to go in future studies.

## WHERE ARE WE NOW?

### Monoclonal antibodies directed against VEGF

*Bevacizumab* is a humanised monoclonal VEGF antibody with antiangiogenic and antitumour activity in preclinical models. Phase I studies exploring different intravenous (i.v.) administration schedules showed that the agent, given as single agent or in combination with various cytotoxic agents, was safe and did not cause dose-limiting toxicity (DLT) when given at predefined doses. Some bleeding episodes were seen that were not attributed to the agent. Antiangiogenic activity was assessed by contrast-enhanced dynamic magnetic resonance imaging (DEMRI), showing a decrease in tumour microvascular permeability. Biological activity was also demonstrated by a decline in serum levels of unbound VEGF (Gordon *et al*, 2001; Margolin *et al*, 2001; Langmuir *et al*, 2002).

Phase II studies in patients with various tumour types showed partial tumour regressions and/or increased time to progression (compared to placebo or cytotoxic treatment alone), when bevacizumab was given in various doses and different dosing intervals, either as single agent or in combination with various cytotoxic agents. In these studies, the increased incidence of thromboembolic complications, bleeding episodes (some of which were lethal), hypertension and proteinuria was the reason for concern (Burnstein *et al*, 2002; Miller *et al*, 2002; Yang *et al*, 2002; Kabbinavar *et al*, 2003). Two randomised phase III studies combining bevacizumab with standard cytotoxic treatment in patients with metastatic breast cancer showed disappointing results (Miller *et al*, 2002,^2003^), but a recently presented randomised phase III study in 800 previously untreated patients with metastatic colorectal cancer comparing treatment with irinotecan, 5-fluorouracil and leucovorin (IFL) and IFL combined with bevacizumab given every 2 weeks showed increased response rates, prolonged time to progression and significantly increased survival with no increased incidence of thromboembolic complications (Hurwitz *et al*, 2003).

*VEGF-trap* is a specific antagonist that binds to and subsequently inactivates VEGF. It is claimed to have a higher affinity for VEGF than monoclonal antibodies, and can be administered subcutaneously. Phase I studies are ongoing (Dupont *et al*, 2003).

### VEGFR-2 antibodies

As ligand–receptor interaction of VEGF and VEGFR-2 is crucial for endothelial proliferation, it is conceivable that apart from blocking VEGF as the principal ligand, blocking VEGFR-2 is another rational approach to prevent this cascade. However, monoclonal antibodies targeting VEGFR-2 have not yet been tested extensively and, to date, only one phase I study with such a compound, IMC-1C11, has been published. No major drug-related toxicity was seen in this small study (Posey *et al*, 2003).

### VEGF receptor tyrosine kinase inhibitors

*SU5416* showed antiangiogenic and antitumour activity in preclinical studies, and was the first VEGF receptor tyrosine kinase inhibitor to be tested clinically (Stopeck *et al*, 2003).

Phase I trials yielded headache and vomiting as DLT. Due to a short half-life, SU5416 had to be given frequently in order to achieve active plasma concentrations for prolonged periods of time. The twice-weekly treatment schedule was studied most often, but even this schedule yielded active plasma concentrations for only a limited period of time. A more frequent intravenous administration schedule was considered to be unpractical, and limited bioavailability hampered the development of an oral compound. Although some clinical activity was recorded, and DEMRI analysis revealed a reduction in tumour vascular permeability, biological activity could not be demonstrated by means of consistent changes in the serum markers of endothelial cell proliferation. In phase I and II combination trials, SU5416 could be given safely in combination with 5-FU and leucovorin, but combining SU5416 with cisplatinum and gemcitabine yielded an unexpectedly high number of severe thromboembolic complications, leading to premature closing of the study (Kuenen *et al*, 2002a). Randomised phase III studies of SU5416 with 5-FU/leucovorin and 5-FU/leucovorin/irinotecan in patients with metastatic colorectal carcinoma failed to show a survival benefit of the SU5416 containing regimens, resulting in the cessation of further development of this compound.

*SU 6668* is an orally available inhibitor of VEGF, bFGF and PDGF receptor tyrosine kinase activity. Phase I trials showed good tolerability with once daily dosing, but a twice daily administration schedule yielded DLT consisting of fatigue, dyspnea, chest pain and pericardial effusions (Rosen *et al*, 2001; Brahmer *et al*, 2002). Pharmacokinetic analysis revealed a short plasma half-life, with plasma concentrations exceeding those that inhibited tyrosine kinase activity in preclinical models in the first day, but not following prolonged administration. Due to these disappointing results, and despite the fact that some clinical activity was noted in clinical studies, the compound was withdrawn from further development (Britten *et al*, 2002; Kuenen *et al*, 2002b; Ueda *et al*, 2002).

*SU11248* is a broad-spectrum orally available tyrosine kinase inhibitor, inhibiting VEGF, PDGF, c-Kit and Flt-3 kinase activity (Mendel *et al*, 2002). This inhibitory spectrum makes this agent to be more than a specific antiangiogenic agent. The recently presented phase I trials exploring either 14 or 28 days treatment followed by 14 days off treatment yielded DLT consisting of fatigue, lethargy and myelosuppression. Two patients died due to drug-related neutropenic sepsis. Tumour regressions have been noted, and positron emission tomography (PET) imaging studies and a rise in serum VEGF levels parallelled by a consistent decrease in soluble VEGFR-2 levels indicate antiangiogenic activity (Manning *et al*, 2003; Raymond *et al*, 2003; Rosen *et al*, 2003; Toner *et al*, 2003). Phase II trials are being initiated.

*PTK787/ZK22854* is an orally available VEGFR-1 and VEGFR-2 tyrosine kinase inhibitor. Ataxia, vertigo and hypertension were noted as DLTs, whereas some cases of venous thromboembolism were recorded (Yung *et al*, 2002). Biological activity has been demonstrated as DEMRI analysis showed a dose-dependent decrease in blood flow in hepatic metastases of colorectal cancer patients (Thomas *et al*, 2001; Drevs *et al*, 2002; Yung *et al*, 2003). Phase II and III trials with PTK in combination with 5-fluorouracil, irinotecan and/or oxaliplatin in patients with metastatic colorectal cancer have been performed or are currently ongoing (Steward *et al*, 2003; Trarbach *et al*, 2003).

*ZD6474* inhibits both VEGF and epidermal growth factor (EGF) receptor tyrosine kinase activity. Following prolonged continuous oral administration, DLT consisted of diarrhoea, hypertension, hepatic toxicity and cutaneous rash, whereas asymptomatic QTc prolongation was seen throughout the dose levels studied (Hurwitz *et al*, 2002; Minami *et al*, 2003). Hints of clinical activity were recorded in some patients. It is conceivable that diarrhoea and skin rash are due to the inhibition of the EGF receptor tyrosine kinase activity, illustrating the broad range of tyrosine kinase inhibition by this agent.

*CP-547,632* is a selective VEGFR-2 receptor tyrosine kinase inhibitor that is currently in phase I studies (Tolcher *et al*, 2002).

### Naturally occurring inhibitors of endothelial cell proliferation

*Thrombospondin-1* is a naturally occurring inhibitor of endothelial cell proliferation. As TSP-1 is a large protein, restricting its pharmacological use, and as its antiangiogenic effects are restricted to the N-terminal region, various structural modifications have been made, leading to TSP-1 mimetic proteins. ABT-510 is such a protein that can be administered subcutaneously. Phase I studies have shown excellent tolerability at all dose levels tested, also following prolonged administration (De Vos *et al*, 2002; Gordon *et al*, 2003). Pharmacokinetic analysis has shown that plasma concentrations exceeding those inhibiting angiogenesis in preclinical studies could be maintained for prolonged periods of time following once or twice daily administration. Phase I combination studies are ongoing, whereas single-agent phase II studies in NSCLC and renal carcinoma have been initiated.

*Endostatin and angiostatin* are both naturally occurring angiogenesis inhibitors that are able to induce the apoptosis of endothelial cells and to inhibit endothelial cell migration and proliferation. Endostatin is a 20 kDa fragment derived from the C-terminal region of collagen XVIII, angiostatin is a 38 kDa fragment of plasminogen. Various mechanisms of action are considered for these antiangiogenic properties.

Phase I studies with recombinant human endostatin given as daily i.v. bolus injections revealed no drug-related toxicity and some hints of clinical activity. The pharmacokinetic analyses showed that, at the highest dose that could be practically administered, drug exposure was lower than that yielding maximum tumour growth inhibition in preclinical studies. Additionally, DEMRI analysis done in a number of patients did not show any relevant changes in perfusion throughout the treatment period (Eder *et al*, 2002) and analysis of various serum markers of angiogenesis was inconclusive (Herbst *et al*, 2002). Currently, studies exploring continuous intravenous and subcutaneous administration of endostatin are being performed, with preliminary data showing increased drug exposure when compared to bolus administration (Hansma *et al*, 2002; Tran *et al*, 2002).

Angiostatin is currently undergoing phase I clinical trials, exploring both i.v. and twice daily subcutaneous injections (Beerepoot *et al*, 2001; DeMoraes *et al*, 2001; Voest *et al*, 2002).

### Other inhibitors of endothelial cell proliferation

*TNP-470* is a derivative of the natural compound fumagilin that showed antiangiogenic activity in preclinical models through inhibition of cyclin-dependent kinase 2, retinoblastoma protein phosphorylation and methionine aminopeptidase.

The first clinical studies with TNP-470 explored weekly 1-h infusions, but as the terminal plasma half-life turned out to be only minutes, additional studies with either more frequent administrations or more protracted infusion schedules (up 120 h) were performed. Dose-limiting toxicity consisted of neuropsychiatric effects (Bhargava *et al*, 1999; Stadler *et al*, 1999; Logothetis *et al*, 2001). More recently, TNP-470 administered as a protracted infusion in combination with paclitaxel and carboplatin has yielded interesting efficacy data in patients with NSCLC (Blumenschein *et al*, 2002).

Although the exact mechanism of antiangiogenic action of *thalidomide* has not been fully elucidated, a large number of clinical studies with this compound have been performed. In particular, patients with multiple myeloma seem to benefit from thalidomide, although some responses have also been recorded in solid tumours. Obstipation, lethargy and peripheral neuropathy have been ascribed to thalidomide.

*Vitaxin* is a monoclonal antibody of the integrin *α*v*β*3 that can be administered subcutaneously. A phase I trial showed good clinical tolerability and antitumour activity in a patient with leiomyosarcoma. Direct antiangiogenic activity was not demonstrated (Gutheil *et al*, 2000). A phase II study in patients with leiomyosarcomas did not confirm antitumour activity (Patel *et al*, 2001).

*Medi-522* is a novel humanised monoclonal antibody targeting the integrin *α*v*β*3 receptor that is currently undergoing phase I studies (Faivre *et al*, 2003).

*Cilengitide* is a small molecule inhibitor of the integrins *α*v*β*3 and *α*v*β*5. A phase I study exploring a continuous twice weekly i.v. administration schedule showed excellent clinical tolerability with no drug-related side effects following a predefined dose escalation schedule (Eskens *et al*, 2003). Pharmacokinetic analysis demonstrated that plasma concentrations exceeding those that yielded optimal antiangiogenic activity in preclinical models were reached. Currently, aditional pharmacodynamic analysis to explore biological activity is being done (Holden *et al*, 2002).

### Vascular targeting agents

*Combretastatin prodrug A4* (*CA4P*) is a tubulin-depolymerising VTA that is able to induce a rapid change in endothelial cell shape, causing a disruption of the endothelial layer, vascular congestion and loss of blood flow. Preclinical studies showed selectivity for rapidly proliferating tumour endothelium, leaving mature endothelial cells unaffected. Dose-limiting toxicity in three phase I studies, exploring different treatment schedules, consisted of acute coronary syndrome, tumour pain and ataxia. QTc prolongation, cutaneous flushing and hot flashes and diffuse abdominal pain and tumour pain were associated with the drug infusion. One pathological complete response lasting 33 months was recorded in a patient with an anaplastic thyroid carcinoma.

Reversible decreases of tumour blood flow were observed using either dynamic enhanced MRI scanning or PET scanning (Stevenson *et al*, 2000; Rustin *et al*, 2001; Dowlati *et al*, 2002).

*AVE8062A* is a combretastatin analogue that is undergoing phase I testing. Transient and reversible myocardial and central nervous system ischaemia were recorded at the highest dose levels studied (Tolcher *et al*, 2003).

*ZD6126* binds to endothelial tubulin, and is able to induce changes in endothelial shape, leading to vascular shutdown. Preclinical studies showed that only immature endothelial cells responded to ZD6126, whereas mature endothelial cells were not affected.

In a phase I study exploring a 10-min single i.v. administration once every 21 days, abdominal pain and gastrointestinal toxicity were dose limiting. Significant changes in tumour blood flow were observed, as some cases of prolonged stable disease (DelProposto *et al*, 2002; Gadgeel *et al*, 2002). A second phase I study is exploring a weekly administration schedule (Radema *et al*, 2002).

*DMXAA* is a flavonoid that, in preclinical models, is able to induce TNF-*α* production and induce vascular disruption, selectively in the tumour microenvironment.

In a phase I trial, DLT consisted of urinary incontinence, various seemingly central nervous system-related toxicities, dyspnea and cardiac toxicity (Rustin *et al*, 2003).

Biological activity was assessed by means of DEMRI analysis, showing changes in tissue perfusion, vessel permeability and vessel surface area (Galbraith *et al*, 2002).

## WHERE ARE WE GOING?

The first clinical studies with antiangiogenic agents were initiated 5 years ago, when preclinical studies in mice showed tumour disappearance and prolonged survival following the administration of endostatin and angiostatin. Scientists and especially the lay press were excited over these results.

Clinical studies of these 5 years have shown that many angiogenesis inhibitors can be given safely to patients. Some antiangiogenic agents were even completely devoid of toxicity, and defining an optimal dose for subsequent activity testing of these agents was difficult, if not impossible. In the meantime, it became obvious that antiangiogenic activity of some agents occurred at very low doses, even more questioning the concept of dose escalation until toxicity. Determining optimal biological activity became an important new end point in phase I studies.

How can we optimally define the optimal biologically effective dose, and which pharmacodynamic analysis should be used for this?

Target inhibition, dormancy of endothelial cells or vascular shutdown with subsequent tumour ischaemia are important biologial end points that have the potential to predict antitumour activity, but the only reliable source to determine these end points is the tumour. Here we are facing practical problems, because most physicians consider taking repeated tumour biopsies to be very cumbersome for patients. Although some authors consider taking these biopsies repeatedly to be feasible (Dowlati *et al*, 2001), the number of studies incorporating these procedures is still small. Although many alternative procedures and strategies have been developed to circumvent this problem, it is conceivable that, in future studies, exploring antiangiogenic agents taking tumour biopsies will become mandatory.

While trying to demonstrate biological activity in a less invasive manner, various surrogate markers of antiangiogenic or antivascular activity have been studied. The analysis of serum markers of endothelial cell proliferation thus far has failed to show consistent changes following treatment with most antiangiogenic agents, but changes in serum concentrations of VEGF and soluble VEGFR-2 seem to have more potential to predict tumour hypoxia (Drevs *et al*, 2003; Toner *et al*, 2003). The pathophysiological mechanisms that explain these changes are still a matter of debate, however, as tumour hypoxia as such has not been demonstrated in human tumours.

Noninvasive techniques such as DEMRI and PET scans are increasingly used to demonstrate the biological activity of antiangiogenic agents or VTAs. These techniques can show changes in tumour blood flow and tumour viability, respectively. In numerous studies, DEMRI and PET scan studies were able to demonstrate these pharmacodynamic effects, but it must be realised that both techniques are still unvalidated to predict antitumour activity or patient benefit. Only large clinical studies correlating the outcomes of DEMRI and PET scan studies with clinical outcome can definitely assess the role of these techniques in future patient management.

Another method to assess the biological activity of antiangiogenic agents is by the use of pharmacokinetic studies. Drug exposure or peak plasma concentrations can be used to determine the dose that is most likely to give antiangiogenic or antitumour activity, but here it has to be realised that comparing human plasma concentrations to target inhibitory concentrations in preclinical models cannot and will not always be predictive for the same biological effect. Nevertheless, pharmacokinetic analysis will remain of crucial importance to prevent unnecessary dose escalation in case of incomplete or saturated absorption with maximal drug exposure, and to show the relations between clinical toxicity and plasma concentrations.

Antiangiogenic agents are cytostatic agents that are not likely to cause tumour regressions in patients with advanced or metastatic cancer. In order to assess their antitumour activity in phase II studies, combining these agents with cytotoxic anticancer agents seems to be a logical step. Clinically, the combination of two antiangiogenic agents, each having a distinctive inhibitory mechanism of action, has not been tested thus far. A large number of studies combining cytotoxic and antiangiogenic agents have been performed. These phase II studies most often had a randomised design, comparing the response rates or time to disease progression. In order to define safe combination dosages, these studies were always preceded by phase I combination studies showing the safety of these combinations.

As some of these randomised phase II trials were indeed able to demonstrate improved antitumour activity of the combination treatment, results of large randomised phase III trials were eagerly awaited. However, until recently, these phase III trials, mostly done in patients with metastatic colorectal or breast cancer, all failed to show improved survival, and serious concern was growing as to whether we would ever be able to demonstrate the clinical efficacy of any of these exciting and promising agents.

It was with great excitement, therefore, that recently the first randomised phase III trial in patients with metastatic colorectal cancer showed improved survival when cytotoxic treatment was combined with bevacizumab. It is this trial that, on the one hand, has reconfirmed the conviction that we, currently, are testing effective new agents, whereas, on the other hand, it has become clear that the only way to assess clinical benefit or a complete generation of these agents will be through these large, costly and time-consuming randomised trials. Besides, we have to accept that more than one randomised trial will be needed, as at this moment it is not even fully clear that the dose tested in this study is in fact the most optimal one. Another intriguing question that remains is why the addition of bevacizumab was beneficial to this group of colorectal carcinoma patients, whereas results of randomised studies with various other antiangiogenic agents in a comparable population of patients all turned out to be negative.

If one is to consider the currently perceived limited antitumour activity of single-agent antiangiogenic treatment, it can be argued that a combination of antiangiogenic agents with different targets or a combination of antiangiogenic agents and VTAs might show increased activity. Preclinical studies exploring such combinations are now being performed, and some promising data have been presented. In addition to this, it is also conceivable that combining antiangiogenic agents with other targeted agents should be explored, but for this the help of pharmaceutical companies who are willing to share their compounds and knowledge is urgently needed.

If one is still to explore single agent antiangiogenic treatment, a situation of minimal residual disease following either cytoreductive surgery or cytotoxic treatment seems most appropriate to adequately assess the role of these agents in secondary cancer growth prevention. These adjuvant studies will, by their design, definitely be time and patient consuming, and here an important as yet unanswered question is that of patient convenience and optimal duration of treatment.

In conclusion, 5 years of clinical studies with antiangiogenic agents have yielded promising results, and therefore antiangiogenic agents have the potential to play a role as active anticancer agents. To definitely confirm this role, specifically designed phase I studies, using the correct end points, must be able to define one or more safe and biologically active doses that can be tested in properly designed randomised phase II trials. Here, a wide array of possible combinations can be thought of. Finally, it is mandatory that in more than one randomised phase III trial patient benefit is demonstrated.

The role of the VTAs is not yet established as only phase I studies have been performed so far, but considering their mechanism of action, more studies will definitely follow.

After a period of flawing interest, antiangiogenic agents have regained their place in the centre of investigational antitumour treatment, and it is without any doubt that in the future their role in clinical practice will be established.
